# Socioeconomic inequalities in low back pain among older people: the JAGES cross-sectional study

**DOI:** 10.1186/s12939-019-0918-1

**Published:** 2019-01-21

**Authors:** Takaaki Ikeda, Kemmyo Sugiyama, Jun Aida, Toru Tsuboya, Nanae Watabiki, Katsunori Kondo, Ken Osaka

**Affiliations:** 1 Department of Rehabilitation, Physical Therapy, Sendai Seiyo Gakuin Junior College, Sendai, Japan; 2 Department of International and Community Oral Health, Tohoku University Graduate School of Dentistry, 4-1, Seiryo-machi, Aoba-ku, Sendai, Miyagi 980-8575 Japan; 3 Sendai Pain Clinic Center, Sendai, Japan; 4 Center for Preventive Medical Sciences, Chiba University, Chiba, Japan; 5 Center for Gerontology and Social Science, National Center for Geriatrics and Gerontology, Obu, Japan

**Keywords:** Income, Educational attainment, Subjective economic situation, Occupation, Low back pain, Socioeconomic status, Health inequalities

## Abstract

**Background:**

Low back pain is an important public health issue across the world. However, it is unclear whether socioeconomic status (SES) is associated with low back pain. This study determines an association between SES and low back pain among older people.

**Methods:**

We used cross-sectional data derived from the year 2013 across 30 Japanese municipalities. The survey was conducted between October 2013 to December 2013. Functionally independent community-dwelling older adults aged 65 and above (*n* = 26,037) were eligible for the study. Multilevel Poisson regression analysis with a robust variance estimator was used to examine the association between SES and low back pain. Self-reported low back pain in the past year was used as a dependent variable. Educational attainment, past occupation, equivalized household income, wealth, and subjective economic situation represented SES and were separately analyzed as independent variables, adjusted for covariates including age and sex.

**Results:**

The prevalence of low back pain was 63.4%. Overall, lower SES were more likely to suffer from low back pain compared with that for the highest. First, as for the educational attainment, the prevalence ratio (PR) (95% credible interval (CI)) for the lowest level was 1.07 (1.02–1.12). Second, as for the past occupation, the PR (95% CI) for the blue-collared workers compared with professionals was 1.06 (1.01–1.11). Third, as for the equalized household income, the PRs (95% CI) for lower middle and the lowest income levels were 1.08 (1.02–1.13) and 1.16 (1.10–1.23), respectively. Fourth, as for the wealth, the PRs (95% CI) for lower middle and the lowest wealth levels were 1.11 (1.04–1.19) and 1.18 (1.11–1.27), respectively. Fifth, as for the subjective economic situation, the PRs (95% CI) for lower middle and the lowest financial conditions were 1.18 (1.10–1.26) and 1.32 (1.22–1.44), respectively.

**Conclusions:**

Significant socioeconomic inequalities were observed in low back pain among older individuals in Japan. Policymakers and clinicians must understand the nature of these inequalities.

**Electronic supplementary material:**

The online version of this article (10.1186/s12939-019-0918-1) contains supplementary material, which is available to authorized users.

## Introduction

Low back pain is the number one cause of disability [1]. It is also most commonly experienced among musculoskeletal pains for all age groups [2, 3]. As a whole, musculoskeletal pains impact individuals’ diseases and functional status such as depression [4], dementia [5], falls [6], and disability [5]. Based on the systematic review of the prevalence of low back pain in the adult populations, the estimated one-year prevalence was 38.0% ± 19.4% and more likely to be higher in the older populations [2].

Socioeconomic inequalities in health among older populations have emerged as a global concern [7, 8]. Recent studies have reported that such inequalities were observed not only in diseases but also in symptoms, including musculoskeletal pains [9–12]. Various studies reported socioeconomic inequalities in the risk factors of low back pain [13–16] such as depression [17], obesity [18], and smoking [18]. However, the results of previous studies on socioeconomic status (SES) and low back pain have been inconsistent. A recent large-scale cross-sectional study from the United States reported that the lowest income levels are significantly associated with low back pain compared with the highest income levels [12]. On the other hand, another cross-sectional study from France reported that there was no association between educational attainment and low back pain [19]. The difference in results might be explained by the different aspects of SES indicators; income is a proxy of the present SES and education is a proxy of the past SES. Seldom studies have investigated the associations between various SES factors and low back pain. Here, we conducted a cross-sectional study to determine the association of past and present SES with low back pain among older Japanese people.

## Methods

### Study population

**Fig. 1:**
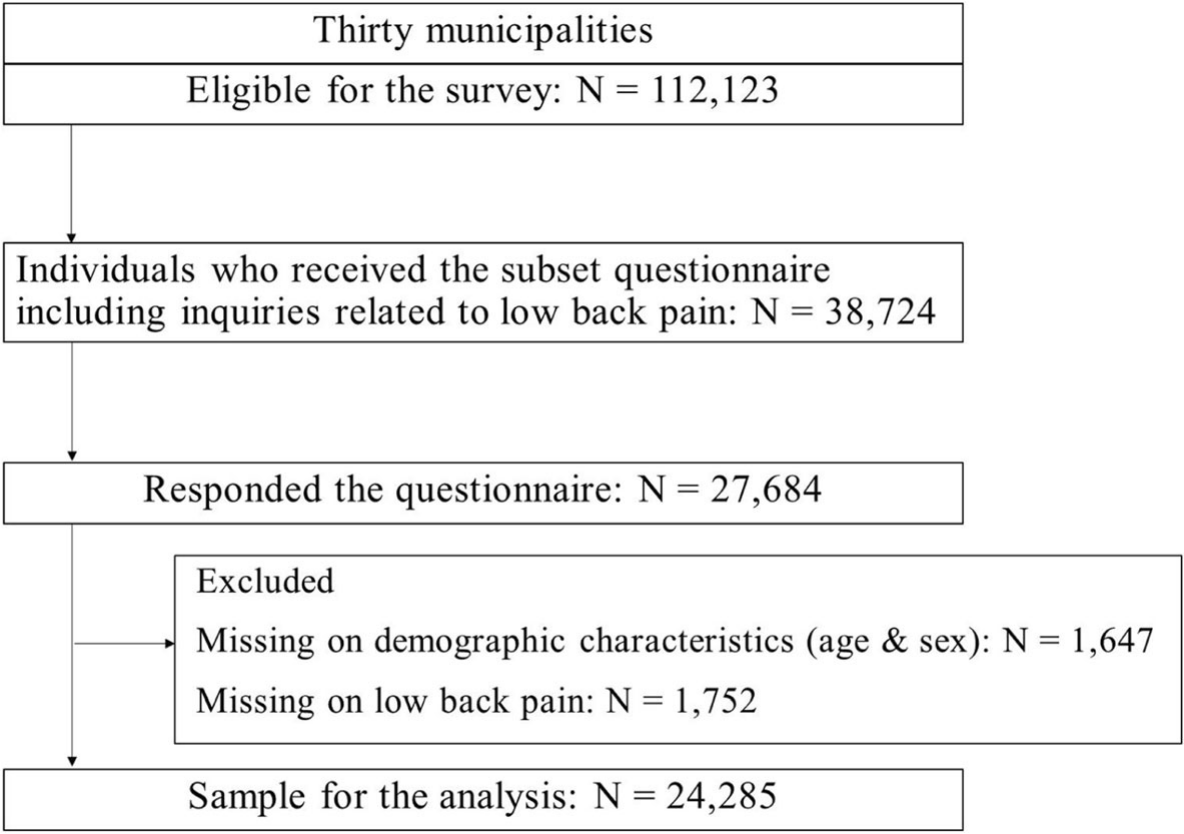
Recruitment diagram of the complete data

We used data from the Japan Gerontological Evaluation Study (JAGES) project, which was cross-sectional data derived from the year 2013. Self-reported questionnaires were mailed to 112,123 people aged ≥65 years, who were not part of the long-term care insurance system [20]. Based on official residential registers obtained from respective municipal governments, the questionnaires were randomly mailed to residents selected from the 17-city areas, all of which have larger populations. In the other 13 municipalities, all of which have a smaller population, questionnaires were mailed to all eligible residents. The survey was conducted between October 2013 and December 2013. The questionnaires were divided into five subsets because many items were inquired as the whole questionnaire. The 112,123 eligible individuals were each distributed one of the five questionnaire subsets. Therefore, 38,724 individuals were mailed the questionnaire that included questions on low back pain. Of them, 27,684 individuals responded, with a response rate of 71.5%. Consequently, we used the data from 24,285 individuals in the analysis (see Fig. 1).

### Dependent variables: Low back pain in the past year

To measure chronic pain rather than acute one, the most widely used period is one-year prevalence of low back pain in previous studies [2]. Although a previous cohort study targeted at older people examined the association between pain intensity within one month and incident disability [5], we consider that such relatively acute pain might not have been enough when considering the long-term mechanism of disability. Therefore, we used one-year prevalence of low back pain. Information on low back pain was obtained by asking the following question to the participants: “Have you felt pain in or around your lower back in the past year?” Responses of “yes” indicated the presence of low back pain. If individuals responded “yes” in the previous question, we further obtained information on the severity of low back pain by asking the following question: “Have you felt physically limited in your daily life because of low back pain?” Responses of “yes” indicated the presence of intense low back pain in our study. Furthermore, we obtained information on medical access for low back pain by asking the following question: “Have you consulted a doctor for low back pain?” with possible answers of “yes” or “no.”

### Independent variables: Socioeconomic status

We assessed five SES indicators as independent variables: Educational attainment, past occupation, equivalized household income, subjective economic situation, and wealth. For older adults, SES can be divided into past or present SES. Thus, we firstly examined the maximum likelihood method with Promax rotations for factor analysis to detect the type of SES indicators. Using factor analysis, educational attainment and past occupation were categorized into past SES. On the other hand, equivalized household income, wealth, as well as subjective economic situation were categorized into present SES (Additional file 1: Table S1). Educational attainment, representing past SES, was categorized into three groups: < 10 years (junior high school; lower secondary education), 10–12 years (high school; upper secondary education), and ≥ 13 years (college or university) [21]. Past occupation, representing past SES, was ascertained by the type of occupation in which participants had been engaged for the longest period. Occupations were categorized as follows: professionals, white-collared workers except for professionals, blue-collared workers, and those who had never worked before. These categories referred to a previous study [22]. We classified equivalized household incomes per year, as a present SES, into four groups: < 1,000,000 yen, 1,000,000–1,999,999 yen, 2,000,000–2,999,999 yen, and ≥ 3,000,000 yen. Wealth, as a present SES, was ascertained as household assets including savings, real estate (e.g. house, land, condominium), stocks, golf membership and was classified into five groups: < 1,000,000 yen, 1,000,000–4,999,999 yen, 5,000,000–9,999,999 yen, 10,000,000–49,999,999 yen, and ≥ 50,000,000 yen. Subjective economic situation, as a present SES, was ascertained by asking the following question: “Which of the following best describes your feelings against your current financial living conditions as a whole?” with possible answers of “very difficult,” “difficult,” “comfortable,” and “very comfortable.”

### Covariates

We used several covariates on the basis of previous works: age (65–69, 70–74, 75–79, 80–85, and ≥ 85 years), sex, number of people living together (living alone, living with others), marital status [23] (married, widowed, divorced, and never married), musculoskeletal disease, body mass index (BMI) [15] (< 18.5 kg/m^2^, 18.5–24.9 kg/m^2^, 25.0–29.9 kg/m^2^, ≥30 kg/m^2^), drinking habit [24, 25] (current, former, never), smoking [16] (current, former, never), physical activity [26, 27] (≥4 times a week, 2–3 times a week, once a week, 1–3 times a month, a few times a year and rare), and depression [13, 14] (none, mild, severe). Following a previous study [26], physical activity was measured as comprising the frequency of moderately intensive activities such as walking (at a brisk pace), dancing, gymnastics, golf, yard work, and car washing. Drinking habit was ascertained by asking the following question: “Do you drink alcohol?” with possible answers of “current”, “former,” and “never.” The Japanese short version of Geriatric Depression Scale (GDS), consisting of 15 questions, which has been previously reported to be validated as a screening index for major depression, was used to assess the prevalence of depressive symptoms [28, 29]. We classified the participants into three groups: those with non-depressive symptoms (GDS < 5), those with mild depression (GDS of 5–9), and those with severe depression (GDS ≥10) [29].

### Statistical analysis

Considering the hierarchical structures of municipalities compared to individuals, multilevel Poisson regression analysis with a robust variance estimator was used to examine the association between SES and low back pain [30]. The data was structured as two levels: individual as level 1 and municipality as level 2. Estimates were obtained from Bayesian estimation using Markov Chain Monte Carlo (MCMC) methods. To avoid multicollinearity, the variables of educational attainment, past occupation, equivalized household income, subjective economic situation, and wealth were separately analyzed in different models, adjusting for the covariates. Dummy variables for all covariates were appropriately added to the models.

We built four regression models: Model 1, a crude model; Model 2, with age and sex adjusted to Model 1; Model 3, with number of persons living together, marital status, musculoskeletal disease, BMI, drinking habit, smoking, physical activity added to Model 2; Model 4, with depression added to Model 3. Depression was considered to be a possible intermediate factor in our analyses based on previous studies [31, 32]. To evaluate the social gradient of pain, we also examined P for trends for each model. Stratified analyses were also performed regarding sex and age (< 75 years old, ≥75 years old), with reference to previous studies [2, 12]. This stratification of age was made for the following reason: copayment for medical services differs between older adults aged < 75 years and ≥ 75 years in Japan; thus, medical access to low back pain might have been affected by economic reasons [33]. Moreover, we performed the same regression analysis in which SES indicators concurrently analyzed in the same model: Model A, past SES indicators (educational attainment and past occupation) concurrently added; Model B, present SES indicators (income, subjective economic situation and wealth) concurrently added; Model C, all SES indicators concurrently added. All of the previously mentioned models were adjusted for age, sex, number of persons living together, marital status, musculoskeletal disease, BMI, drinking habit, smoking and physical activity.

Before performing regression analyses, we employed multiple imputation under the missing at random (MAR) assumption to handle the problem of missing values. Missing variables were imputed by multivariate imputation chained equations (MICE) using following variables; sex, age, equivalized household income, educational attainment, past occupation, wealth, subjective economic situation, number of people living together, marital status, presence of knee pain, presence of low back pain, smoking, drinking habit, BMI, GDS, physical activity, and residential municipality. On the basis of a previous work, we imputed not only independent variables and covariates, but also dependent variable [34]. Rubin’s rule was used to combine the results across 10 imputed datasets [35]. For the complete case analysis, we used listwise deletion methods.

For sensitivity analysis, we performed the same analysis among participants who subjectively reported physical limitation due to low back pain in daily life (*n* = 7878) [36], as intense low back pain might shorten healthy life expectancy [37]. Multilevel analyses were performed with MLwiN, version 3.02 (Centre for Multilevel Modelling, University of Bristol) via Stata, version 15.1 (Stata Corp, College Station, TX). All other analyses were conducted using Stata.

## Results

### Demographic characteristics

Table 1 and Additional file 1: Table S2 summarize the demographic characteristics, health status, and health behaviors of all eligible participants, respectively. The prevalence of low back pain in the past year was 63.4% in the complete data. (Additional file 1: Table S2) Those who were older, female, living alone, less educated, lower income level and with lower wealth were more likely to suffer from low back pain in the past year (Table 1).

**Table 1:** The presence of low back pain in participants by characteristic (*n* = 26,037)

Characteristics	Total	Having low back pain	*P*-value
	N	N (%)	
Sex			
Male	12,088	6892 (61.0)	<.01
Female	13,949	8509 (65.5)	
Age, years	74.02 (6.25) ^a^		
65–69	7220	4155 (60.5)	<.01
70–74	7796	4479 (61.3)	
75–79	5754	3439 (64.8)	
80–84	3519	2229 (69.0)	
≥ 85	1748	1099 (69.8)	
Educational attainment, years			
< 10 (junior high school; lower secondary education)	10,847	6602 (66.0)	<.01
10–12 (high school; upper secondary education)	9509	5597 (62.4)	
≥ 13 (college or university degree)	5072	2866 (59.3)	
Past occupation			
Professionals	3742	2179 (59.4)	<.01
White-collared workers	5305	3150 (60.4)	
Blue-collared workers	9627	6010 (64.3)	
Never worked before	1302	826 (65.5)	
Equivalized household income, yen			
< 1 million	3119	2107 (70.3)	<.01
1 million–1.99 million	7735	4795 (63.7)	
2 million–2.99 million	4819	2839 (60.3)	
≥ 3 million	4974	2863 (58.6)	
Subjective economic situation			
Very difficult	1963	1378 (76.0)	<.01
Difficult	8853	5556 (67.2)	
Comfortable	12,247	6871 (59.8)	
Very comfortable	2286	1225 (56.5)	
Wealth, yen			
< 1 million	2101	1428 (70.3)	<.01
1 million–4.99 million	2815	1800 (65.8)	
5 million–9.99 million	3255	1971 (62.0)	
10 million–49.99 million	7842	4693 (61.0)	
≥ 50 million	2828	1586 (57.2)	
Depression			
Non (GDS < 5)	15,592	8578 (58.2)	<.01
Mild (GDS of 5–9)	4178	2834 (71.9)	
Severe (GDS ≥ 10)	1485	1101 (79.6)	

^a^ mean age (SD). Chi-squared test was performed

1 US dollar is approximately 100 yen and 1 EURO is approximately 130 yen

### Back pain and socioeconomic status

Table 2 summarizes the results of multilevel Poisson regression analyses for the five SES independent variables after imputation for missing data. The municipality level variances were small in all models (Table 2).

**Table 2:** The association of each socioeconomic status with low back pain after multiple data imputations (n = 26,037. Multilevel Poisson regression analysis)

Socioeconomic status	Model 1		Model 2		Model 3		Model 4	
	PR	95% CI	PR	95% CI	PR	95% CI	PR	95% CI
Fixed parameter								
Education, years (ref, ≥13)								
10–12	1.05	1.01, 1.10	1.04	0.99, 1.09	1.03	0.99, 1.09	1.03	0.98, 1.08
< 10	1.12	1.07, 1.17	1.09	1.04, 1.14	1.07	1.02, 1.12	1.05	1.002, 1.10
*P* for trend	< 0.01		< 0.01		< 0.01		0.01	
Random part								
Municipality level variance (standard error)	0.001 (< 0.001)		0.001 (< 0.001)		0.001 (< 0.001)		0.001 (< 0.001)	
Past occupation (ref, professionals)								
Fixed parameter								
White-collared workers	1.02	0.97, 1.07	1.00	0.95, 1.06	1.01	0.95, 1.06	1.01	0.95, 1.06
Blue-collared workers	1.08	1.03, 1.14	1.06	1.02, 1.11	1.06	1.01, 1.11	1.04	1.001, 1.10
Never worked before	1.11	1.04, 1.19	1.04	0.97, 1.12	1.03	0.95, 1.12	1.01	0.93, 1.09
*P* for trend	< 0.01		< 0.01		< 0.01		0.02	
Random part								
Municipality level variance (standard error)	0.001 (< 0.001)		0.001 (< 0.001)		0.001 (< 0.001)		0.001 (< 0.001)	
Income, yen (ref, ≥3 million)								
2 million–2.99 million	1.03	0.98, 1.09	1.03	0.98, 1.08	1.03	0.97, 1.09	1.02	0.97, 1.08
1 million–1.99 million	1.09	1.04, 1.14	1.09	1.04, 1.14	1.08	1.02, 1.13	1.05	1.002, 1.11
< 1 million	1.20	1.14, 1.26	1.17	1.11, 1.23	1.16	1.10, 1.23	1.12	1.06, 1.19
*P* for trend	< 0.01		< 0.01		< 0.01		< 0.01	
Random part								
Municipality level variance (standard error)	0.001 (< 0.001)		0.001 (< 0.001)		0.001 (< 0.001)		0.001 (< 0.001)	
Subjective economic situation (ref, very comfortable)								
Fixed parameter								
Comfortable	1.06	1.00, 1.12	1.07	1.004, 1.13	1.05	0.99, 1.12	1.04	0.98, 1.11
Difficult	1.19	1.12, 1.26	1.21	1.14, 1.29	1.18	1.10, 1.26	1.14	1.07, 1.22
Very difficult	1.34	1.25, 1.44	1.36	1.26, 1.47	1.32	1.22, 1.44	1.22	1.11, 1.33
*P* for trend	< 0.01		< 0.01		< 0.01		< 0.01	
Random part								
Municipality level variance (standard error)	0.001 (< 0.001)		0.001 (< 0.001)		0.001 (< 0.001)		0.001 (< 0.001)	
Wealth, yen (ref, ≥50 million)								
Fixed parameter								
10 million–49.99 million	1.07	1.01, 1.12	1.07	1.01, 1.12	1.05	0.99, 1.11	1.04	0.98, 1.10
5 million–9.99 million	1.09	1.03, 1.16	1.09	1.02, 1.15	1.07	1.004, 1.14	1.05	0.98, 1.12
1 million–4.99 million	1.15	1.09, 1.23	1.14	1.08, 1.22	1.11	1.04, 1.19	1.08	1.01, 1.16
< 1 million	1.22	1.15, 1.30	1.21	1.14, 1.29	1.18	1.11, 1.27	1.13	1.06, 1.21
*P* for trend	< 0.01		< 0.01		< 0.01		< 0.01	
Random part								
Municipality level variance (standard error)	0.001 (< 0.001)		0.001 (< 0.001)		0.001 (< 0.001)		0.001 (< 0.001)	

Abbreviations: *PR* prevalence ratio, *95% CI* 95% credible interval

Socioeconomic status was separately added to each model. Model 1, a crude model; Model 2, with age and sex adjusted to Model 1; Model 3, with number of persons living together, marital status, musculoskeletal disease, BMI, drinking habit, smoking and physical activity added to Model 2; Model 4, with depression added to Model 3

First, as for educational attainment, after adjusting for covariates and risk factors (Model 3), those of the lowest educational level were more likely to experience low back pain compared with the highest educational level–a prevalence ratio (PR) (95% credible interval (CI)) of 1.07 (1.02, 1.12). This association was attenuated after additional adjustment for depression–a PR (95% CI) of 1.05 (1.002, 1.10) (Model 4). Second, as with past occupation, the PR (95% CI) of experiencing low back pain for blue-collared workers compared with professionals was 1.06 (1.01, 1.11). This association was attenuated after additional adjustment for depression–a PR (95% CI) of 1.04 (1.001, 1.10) (Model 4). Third, with regard to equivalized household income, after adjusting for covariates and risk factors (Model 3), the PRs (95% CI) for lower middle and the lowest income levels were 1.08 (1.02, 1.13) and 1.16 (1.10, 1.23), respectively. Significant associations persisted after controlling for additional adjustment for depression (Model 4). Fourth, with regard to subjective economic situation, Model 3 showed that the PRs (95% CIs) for the “difficult” and the “very difficult” situations were 1.18 (1.10, 1.26) and 1.32 (1.22, 1.44), respectively. The associations similarly persisted in Model 4. Finally, with regard to wealth, Model 3 showed that PRs (95% CIs) for the lower middle and the lowest wealth levels were 1.11 (1.04, 1.19) and 1.18 (1.11, 1.27), respectively. The associations similarly persisted in Model 4. P for trends in education, past occupation, income, subjective economic situation, and wealth were significant (Table 2). All results using complete data were similar to those from multiple imputation pooled data (Additional file 1: Table S3).

Additional file 1: Table S3 also shows the associations of covariates and risk factors with low back pain. Overall, being older, female, the presence of musculoskeletal pain, obesity, and depression were associated with low back pain in several models. As for BMI, both overweight and obesity were associated with low back pain compared with normal weight (Model 3). For example, when SES was determined by income level, PRs (95% CI) for overweight and obesity were 1.07 (1.02, 1.12) and 1.16 (1.02, 1.32), respectively. The associations persisted after additional adjustment for depression (Model 4). Similarly, as for depression, both mild and severe depression were associated with low back pain compared with non-depression (Model 3). When SES was determined by income level, PRs (95% CI) for mild and severe were 1.19 (1.13, 1.26) and 1.29 (1.19, 1.40), respectively. Additional file 1: Table S4 shows the results of stratified analyses. When considering sex stratification, sex differences were observed regarding education, past occupation, and equivalized household income–the associations were observed among males. When considering age stratification, no clear differences were observed between ≥75 years old and < 75 years old.

Table 3 shows the associations when SES indicators concurrently added to the regression models. For past SES indicators, the significant association of educational attainment persisted in the lowest group. Meanwhile, for past occupation, the association was no longer statistically significant for blue-collared (Model A). For present SES indicators, the significant associations of subjective economic situation persisted among difficult and very difficult, while the other associations were attenuated (Model B). When all SES indicators were included in the model, only the significant association of subjective economic situation being difficult and very difficult persisted, while associations were no longer statistically significant for other indicators.

**Table 3:** The association of combined socioeconomic status with low back pain after multiple data imputations (*n* = 26,037. Poisson regression analysis)

Socioeconomic status	Model A		Model B		Model C	
	PR	95% CI	PR	95% CI	PR	95% CI
Fixed effect parameters						
Educational attainment, years (ref, ≥13)						
10–12	1.03	0.98, 1.08	–	–	1.01	0.97, 1.06
< 10	1.05	1.002, 1.11	–	–	1.02	0.97, 1.08
Past occupation (ref, professionals)						
White-collared workers	1.01	0.95, 1.06	–	–	1.01	0.96, 1.07
Blue-collared workers	1.05	0.99, 1.10	–	–	1.03	0.97, 1.08
Never worked before	1.02	0.94, 1.11	–	–	1.00	0.92, 1.08
Income, yen (ref, ≥3 million)						
2 million–2.99 million	–	–	1.00	0.95, 1.06	1.00	0.95, 1.06
1 million–1.99 million	–	–	1.02	0.96, 1.07	1.02	0.96, 1.08
< 1 million	–	–	1.06	0.99, 1.13	1.06	0.99, 1.14
Subjective economic situation (ref, very comfortable)						
Comfortable	–	–	1.04	0.97, 1.11	1.04	0.97, 1.11
Difficult	–	–	1.12	1.04, 1.20	1.15	1.07, 1.24
Very difficult	–	–	1.17	1.07, 1.29	1.27	1.15, 1.39
Wealth, yen (ref, ≥50 million)						
10 million–49.99 million	–	–	1.01	0.95, 1.07	1.00	0.94, 1.06
5 million–9.99 million	–	–	0.99	0.93, 1.07	0.99	0.92, 1.06
1 million–4.99 million	–	–	1.01	0.94, 1.09	1.00	0.93, 1.08
< 1 million	–	–	1.03	0.95, 1.12	0.99	0.95, 1.11
Random parameter						
Municipality level variance (standard error)	0.001 (< 0.001)		0.001 (< 0.001)		0.001 (< 0.001)	

Abbreviations: *PR* prevalence ratio, *95% CI* 95% credible interval

Model A, educational attainment and past occupation concurrently added to the model adjusting for age, sex, number of persons living together, marital status, musculoskeletal disease, BMI, drinking habit, smoking and physical activity

Model B, income, subjective economic situation, wealth concurrently added to the model adjusting for age, sex, number of persons living together, marital status, musculoskeletal disease, BMI, drinking habit, smoking and physical activity

Model C, all socioeconomic status concurrently added to the model adjusting for age, sex, number of persons living together, marital status, musculoskeletal disease, BMI, drinking habit, smoking and physical activity

For sensitivity analysis, associations were emphasized for all models when performing the same analysis among participants who experienced low back pain with limitations in daily life (*n* = 7878). (see Additional file 1: Table S5).

## Discussion

To the best of our knowledge, our study was the first to reveal the association of past and present SES with low back pain in the older population. We found that participants with low SES, as measured by education, past occupation, income, subjective economic situation, and wealth, were more prone to experience low back pain compared with those with high SES. Moreover, these results showed that there was a socioeconomic gradient in low back pain; people with lower socioeconomic background were more likely to suffer from pain. Therefore, low back pain is a problem for not only of the deprived people, but also a problem for the whole society. Expectedly, the associations of SES with low back pain dramatically attenuated when depression was adjusted for.

Regarding present SES, a cross-sectional study from the United States found lower-income levels to be associated with low back pain in the general population [12]. This study also indicated that associations between income and low back pain were stronger among males than among females [12]. The findings of our study are also in line with those of this cross-sectional study. We found that older individuals with a lower income level were more likely to suffer from low back pain. This association was strongly observed among older males.

We also newly elucidated the association between other present SES, as represented by wealth or subjective economic situation, and low back pain. Accordingly, we found that participants with a lower level of both wealth and subjective economic situation were more likely to experience low back pain, when separately analyzed.

Our further analyses which included all SES factors showed that the impact of more difficult subjective economic situation remained significant while the effects of other SES indicators were attenuated (see Table 3). Recently, subjective economic situation has been focused upon as a new SES indicator representing the perceived relative deprivation of individuals [38, 39]. A cross-sectional study from Germany showed that subjective economic situation mediates associations between objective SES indicators (education, occupation, and income) and depressive symptoms in adults [39]. Moreover, the study reported that the association of subjective economic situation with poor mental health was stronger than that of other SES indicators [39]. Our findings have the same context with these results to show that the subjective economic situation had the largest impact. Furthermore, we revealed that present SES was found to be associated with low back pain among participants aged < 75 years as well as ≥75 years. This indicates that present SES-related inequalities persist throughout the life.

According to our understanding, this study is among the first to reveal the associations of past SES, as measured by educational attainment and past occupation, with low back pain among older individuals. We found that participants with the lowest educational level and blue-collared workers were more likely to suffer from low back pain. Furthermore, the association between education/occupation and low back pain was stronger among males than among females. For educational attainment, in contrast to our study, a cross-sectional study from France that interviewed labor population reported that the association of educational attainment with low back pain was no longer statistically significant when adjusting for several lifestyle indicators, including BMI and smoking [19]. The difference in educational inequalities between studies might be explained as follows: educational inequalities affect health via health literacy [40], and health literacy is significantly higher in labor generations compared with that in older generations [41, 42]. Therefore, such differences between studies emerged due to demographic differences. No previous study has investigated the association of occupational inequalities with low back pain among older populations. However, numerous previous studies have indicated that heavy labor—a common issue faced by many blue-collared workers—is a risk factor of low back pain [43–46]. Our study is in accordance with the results of these prior studies. Similar to present SES, associations of past SES attenuated when all status indicators were mutually adjusted (see Table 2, Model 4). Furthermore, the association of educational attainment with low back pain was also observed among participants aged < 75 years as well as ≥75 years, indicating that educational inequalities persist throughout the life.

When considering the mechanism of low back pain, the role of risk factors must be determined. Previous studies have indicated that depression [13, 14], obesity [15], smoking [16], and lower-income level [12] are risk factors of low back pain, which is partially in accordance with our findings. Consistent with the results of a previous study [12], present SES as represented by income, subjective economic situation, and wealth were found to be statistically associated with low back pain among older adults. Two possible pathways exist for present SES-related inequalities in health: psychosocial stress and material poverty [47]. Subjective economic situation is considered to be a result of income level and is considered to represent psychosocial stress rather than material poverty [47, 48]. Moreover, individuals with lower income levels are more likely to face barriers in accessing medical care [49]. In our study, among participants with low back pain, medical access to low back pain was significantly different by SES (see Additional file 1: Table S6). This indicated that barriers in accessing medical care would be a proxy for material poverty to account for socioeconomic inequalities in low back pain. A previous study indicated a mutual effect between depression and low back pain [14]. Additionally, a causal relation between low SES and depression has been previously reported [31, 32], which supports our idea of depression as an intermediary factor. In addition to depression, numerous earlier studies have reported obesity [15] to be risk factors of low back pain. In our study, overweight and obesity were associated with low back pain. The associations of obesity somewhat attenuated when depression was additionally adjusted for. Previous studies have reported that such adverse health-related factors were strongly related to psychosocial stress [38, 39], derived from relative deprivation. Therefore, in addition to depression, obesity might contribute to low back pain through psychosocial stress that is affected by SES. Furthermore, the association of drinking habit with low back pain was not statistically significant in our study. However, previous studies have indicated that alcohol abuse might be associated with low back pain [24, 25]. We could not identify participants with alcohol abuse; however, alcohol abuse is associated with low SES [50].

There are several strengths and limitations of our study. First, we examined the association of past and present SES with low back pain. Second, we analyzed a large sample size (*n* = 26,037), which is higher than that analyzed in previous studies [12, 19]. The first limitation of our study is that we were unable to distinguish between acute and chronic pain, which leads to regression dilution bias. In contrast to chronic pain, a previous study has shown that individuals with a higher income level were more likely to experience acute low back pain [12]. Hence, we believe that our results are under-estimating the associations when considering such biases. Second, the pain questionnaire we used lacked information on degree of pain. There is a possibility that inequalities in low back pain might differ in degree of pain. In fact, in our sensitivity analysis, the associations were emphasized for all models when performing the same regression analysis among participants who experienced low back pain with limitations in daily life (see Additional file 1: Table S5). Future studies should include question about degree of pain. Third, we could not clarify the causal pathway because this is a cross-sectional study. Thus, the probable mediation by depressive conditions is not always consistent. However, we revealed that past SES and present SES were associated with low back pain. Longitudinal or cohort studies are necessary for future studies. Fourth, our study participants were not disabled and were not eligible for the Japanese long-term care insurance system. Future study is expected to investigate association between SES and low back pain among population including those of highly physically limited older people. Fifth, the generalizability of the present results to the entire Japanese population remains unclear. This is because the 30 municipalities investigated in this study were not randomly selected, and the sampling method for residents differed according to the population of the municipality. It was difficult to compare our study population with the entire older population due to lack of demographic characteristics in national survey.

## Conclusion

We analyzed data from a cross-sectional study, revealing that socioeconomic inequalities were significantly associated with low back pain among the older Japanese population. Policymakers and clinicians must understand the nature of these inequalities.

## Additional file

Additional file 1: **Table S1.** Factor loadings of each socioeconomic status. **Table S2.** Health status and health behaviors of all eligible participants (*n* = 26,037). **Table S3.** The associations of each parameter with low back pain in complete data (*n* = 24,285. Multilevel Poisson regression analysis). **Table S4.** The association of socioeconomic status with low back pain, stratified by sex or age after multiple data imputations (*n* = 26,037. Multilevel Poisson regression analysis). **Table S5.** The association of socioeconomic status with *severe* low back pain, stratified by sex or age in the complete dataset (*n* = 16,762. Separately Multilevel Poisson regression analysis). **Table S6.** Differences in medical access for low back pain among participants having low back pain by socioeconomic status (*n* = 15,401). (DOCX 119 kb)

## Abbreviations

BMI: Body mass index
CI: Credible interval
GDS: Geriatric Depression Scale
JAGES: Japan Gerontological Evaluation Study
PR: Prevalence ratio
SES: Socioeconomic status

## References

[1] Vos T, Allen C, Arora M, Barber RM, Brown A, Carter A (2016). Global, regional, and national incidence, prevalence, and years lived with disability for 310 diseases and injuries, 1990–2015: a systematic analysis for the global burden of disease study 2015. Lancet.

[2] Hoy D, Bain C, Williams G, March L, Brooks P, Blyth F (2012). A systematic review of the global prevalence of low back pain. Arthritis Rheum.

[3] Meucci RD, Fassa AG, Xavier Faria NM (2015). Prevalence of chronic low back pain: systematic review. Rev Saude Publica.

[4] Gerrits MMJ, van Oppen P, van Marwijk HWJ, Penninx BWJH, van der Horst HE (2014). Pain and the onset of depressive and anxiety disorders. Pain.

[5] Kaiho Y, Sugawara Y, Sugiyama K, Tomata Y, Endo Y, Toyama H (2018). Impact of pain on incident risk of disability in elderly Japanese cause-specific analysis. Anesthesiology.

[6] Stubbs B, Binnekade T, Eggermont L, Sepehry AA, Patchay S, Schofield P (2014). Pain and the risk for falls in community-dwelling older adults: systematic review and meta-analysis. Arch Phys Med Rehabil.

[7] Kröger H, Fritzell J, Hoffmann R. The association of levels of and decline in grip strength in old age with trajectories of life course occupational position. PLoS One. 2016;11:e0155954. 10.1371/journal.pone.0155954 PMC4883757 27232696

[8] Kondo N, Sembajwe G, Kawachi I, Van Dam RM, Subramanian SV, Yamagata Z (2009). Income inequality, mortality, and self rated health: meta-analysis of multilevel studies. BMJ.

[9] Sommer I, Griebler U, Mahlknecht P, Thaler K, Bouskill K, Gartlehner G, et al. Socioeconomic inequalities in non-communicable diseases and their risk factors: an overview of systematic reviews. BMC Public Health. 2015;15:914. 10.1186/s12889-015-2227-y PMC4575459 26385563

[10] Etman A, Burdorf A, Van der Cammen TJM, Mackenbach JP, Van Lenthe FJ (2012). Socio-demographic determinants of worsening in frailty among community-dwelling older people in 11 European countries. J Epidemiol Community Health.

[11] Soler-Vila H, García-Esquinas E, León-Muñoz LM, López-García E, Banegas JR, Rodríguez-Artalejo F (2016). Contribution of health behaviours and clinical factors to socioeconomic differences in frailty among older adults. J Epidemiol Community Health.

[12] Riskowski JL (2014). Associations of socioeconomic position and pain prevalence in the United States: findings from the National Health and nutrition examination survey. Pain Med.

[13] Currie SR, Wang J (2005). More data on major depression as an antecedent risk factor for first onset of chronic back pain. Psychol Med.

[14] Meyer T, Cooper J, Raspe H (2007). Disabling low back pain and depressive symptoms in the community-dwelling elderly: a prospective study. Spine.

[15] Zhang T-T, Liu Z, Liu Y-L, Zhao J-J, Liu D-W, Tian Q-B (2018). Obesity as a risk factor for low Back pain. Clin Spine Surg.

[16] Shiri R, Karppinen J, Leino-Arjas P, Solovieva S, Viikari-Juntura E (2010). The association between smoking and low Back pain: a meta-analysis. Am J Med.

[17] Muntaner C, Eaton WW, Miech R, O’Campo P (2004). Socioeconomic position and major mental disorders. Epidemiol Rev.

[18] Shaw BA, McGeever K, Vasquez E, Agahi N, Fors S (2014). Socioeconomic inequalities in health after age 50: are health risk behaviors to blame?. Soc Sci Med.

[19] Leclerc A, Gourmelen J, Chastang JF, Plouvier S, Niedhammer I, Lanoë JL (2009). Level of education and back pain in France: the role of demographic, lifestyle and physical work factors. Int Arch Occup Environ Health.

[20] Tamiya N, Noguchi H, Nishi A, Reich MR, Ikegami N, Hashimoto H (2011). Population ageing and wellbeing: lessons from Japan’s long-term care insurance policy. Lancet.

[21] Amemiya A, Fujiwara T, Murayama H, Tani Y, Kondo K (2017). Adverse childhood experiences and higher-level functional limitations among older Japanese people: results from the JAGES study. J Gerontol Ser A.

[22] Tsuboya T, Aida J, Osaka K, Kawachi I (2015). Working overtime and risk factors for coronary heart disease: a propensity score analysis based in the J-SHINE (Japanese study of stratification, health, income, and neighborhood) study. Am J Ind Med.

[23] Wettstein M, Eich W, Bieber C, Tesarz J (2018). Pain intensity, disability, and quality of life in patients with chronic low Back pain: does age matter?. Pain Med.

[24] Ferreira PH, Pinheiro MB, Machado GC, Ferreira ML (2013). Is alcohol intake associated with low back pain? A systematic review of observational studies. Man Ther.

[25] Leboeuf-Yde C (2000). Alcohol and low-back pain: a systematic literature review. J Manip Physiol Ther.

[26] Pinto RZ, Ferreira PH, Kongsted A, Ferreira ML, Maher CG, Kent P (2014). Self-reported moderate-to-vigorous leisure time physical activity predicts less pain and disability over 12 months in chronic and persistent low back pain. Eur J Pain.

[27] Stubbs B, Binnekade TT, Soundy A, Schofield P, Huijnen IPJ, Eggermont LHP (2013). Are older adults with chronic musculoskeletal pain less active than older adults without pain? A systematic review and meta-analysis. Pain Med.

[28] Burke WJ, Roccaforte WH, Wengel SP (1991). The short form of the geriatric depression scale: a comparison with the 30-item form. J Geriatr Psychiatry Neurol.

[29] Wada T, Ishine M, Kita T, Fujisawa MMK (2003). Depression screening of elderly community-dwelling Japanese. J Am Geriatr Soc.

[30] McNutt LA, Wu C, Xue X, Hafner JP (2003). Estimating the relative risk in cohort studies and clinical trials of common outcomes. Am J Epidemiol.

[31] Zimmerman FJ, Katon W (2005). Socioeconomic status, depression disparities, and financial strain: what lies behind the income-depression relationship?. Health Econ.

[32] Lorant V, Croux C, Weich S, Deliège D, Mackenbach J, Ansseau M (2007). Depression and socio-economic risk factors: 7year longitudinal population study. Br J Psychiatry.

[33] Shi L, Chen C-C, Nie X, Zhu J, Hu R (2014). Racial and socioeconomic disparities in access to primary care among people with chronic conditions. J Am Board Fam Med.

[34] Young R, Johnson D. Imputing the missing Y’s: implications for survey producers and survey users. American Association for Public Opinion Research. 2010;6242–6248.

[35] Rubin DB, Schenker N (1985). Multiple imputation for interval estimation from surveys with ignorable nonresponse. J Am Stat Assoc.

[36] Sun B, Vanderweele T, Tchetgen Tchetgen EJ (2017). A multinomial regression approach to model outcome heterogeneity. Am J Epidemiol.

[37] Hoy D, March L, Brooks P, Blyth F, Woolf A, Bain C (2014). The global burden of low back pain: estimates from the global burden of disease 2010 study. Ann Rheum Dis.

[38] Goodman E, Adler NE, Daniels SR, Morrison JA, Slap GB, Dolan LM (2003). Impact of objective and subjective social status on obesity in a biracial cohort of adolescents. Obes Res.

[39] Hoebel J, Maske UE, Zeeb H, Lampert T. Social inequalities and depressive symptoms in adults: the role of objective and subjective socioeconomic status. PLoS One. 2017;12:e0169764. 10.1371/journal.pone.0169764 PMC5249164 28107456

[40] Van Der Heide I, Wang J, Droomers M, Spreeuwenberg P, Rademakers J, Uiters E (2013). The relationship between health, education, and health literacy: results from the dutch adult literacy and life skills survey. J Health Commun.

[41] Ashida S, Goodman M, Pandya C, Koehly LM, Lachance C, Stafford J (2011). Age differences in genetic knowledge, health literacy and causal beliefs for health conditions. Public Health Genomics.

[42] Beauchamp A, Buchbinder R, Dodson S, Batterham RW, Elsworth GR, McPhee C, et al. Distribution of health literacy strengths and weaknesses across socio-demographic groups: a cross-sectional survey using the health literacy questionnaire (HLQ). BMC Public Health. 2015;15:678. 10.1186/s12889-015-2056-z PMC4508810 26194350

[43] Hestbaek L, Leboeuf-Yde C, Kyvik KO. Are lifestyle-factors in adolescence predictors for adult low back pain? A cross-sectional and prospective study of young twins. BMC Musculoskelet Disord. 2006;7:27. 10.1186/1471-2474-7-27 PMC1464095 16539729

[44] Feldman DE, Shrier I, Rossignol M, Abenhaim L (2001). Risk factors for the development of low back pain in adolescence. Am J Epidemiol.

[45] Mustard CA, Kalcevich C, Frank JW, Boyle M (2005). Childhood and early adult predictors of risk of incident back pain: Ontario child health study 2001 follow-up. Am J Epidemiol.

[46] Mattila VM, Saarni L, Parkkari J, Koivusilta L, Rimpelä A (2008). Predictors of low back pain hospitalization - a prospective follow-up of 57,408 adolescents. Pain.

[47] Kondo N (2012). Socioeconomic disparities and health: impacts and pathways. J Epidemiol.

[48] Schulz AJ, Israel BA, Zenk SN, Parker EA, Lichtenstein R, Shellman-Weir S (2006). Psychosocial stress and social support as mediators of relationships between income, length of residence and depressive symptoms among African American women on Detroit’s eastside. Soc Sci Med.

[49] Elstad JI. Income inequality and foregone medical care in Europe during the great recession: multilevel analyses of EU-SILC surveys 2008-2013. Int J Equity Health. 2016;15:101. 10.1186/s12939-016-0389-6 PMC4936318 27388561

[50] Probst C, Roerecke M, Behrendt S, Rehm J (2014). Socioeconomic differences in alcohol-attributable mortality compared with all-cause mortality: a systematic review and meta-analysis. Int J Epidemiol.

