# Supplementary material for: Socioeconomic inequalities in low back pain among older people: the JAGES cross-sectional study
Source: Int J Equity Health. 2019 Jan 21;18:15. doi: 10.1186/s12939-019-0918-1 (PMC6341699; doi:10.1186/s12939-019-0918-1)
Supplement: Supplementary file 1 — Table S1. Factor loadings of each socioeconomic status. Table S2. Health status and health behaviors of all eligible participants (n = 26,037). Table S3. The associations of each parameter with low back pain in complete data (n = 24,285. Multilevel Poisson regression analysis). Table S4. The association of socioeconomic status with low back pain, stratified by sex or age after multiple data imputations (n = 26,037. Multilevel Poisson regression analysis). Table S5. The association of socioeconomic status with severe low back pain, stratified by sex or age in the complete dataset (n = 16,762. Separately Multilevel Poisson regression analysis). Table S6. Differences in medical access for low back pain among participants having low back pain by socioeconomic status (n = 15,401). (DOCX 119 kb) [file 12939_2019_918_MOESM1_ESM.docx]

**Table S1.** Factor loadings of each socioeconomic status

| Socioeconomic status | Past | Present |
| --- | --- | --- |
| Educational attainment | .66 | -.0003 |
| Past occupation | .42 | .004 |
| Equivalized household income | .09 | .64 |
| Subjective economic situation | -.08 | .73 |
| Wealth | .04 | .57 |

We applied the maximum likelihood method with Promax rotations for factor analysis to confirm type of socioeconomic status (past or present).

**Table S2.** Health status and health behaviors of all eligible participants (n = 26,037)

| Health status/health behaviors | All eligible participants |
| --- | --- |
|  | n (%) |
| Low back pain in the past year |  |
| Yes | 15,401 (63.4) |
| Low back pain *with* limitations in daily life | 7,878 |
| Low back pain *without* limitations in daily life | 7,256 |
| Missing on limitations in daily life | 267 |
| No | 8,884 (36.6) |
| Missing information on low back pain | 1,752 |
| BMI (kg/m^2^) | 22.80 (3.17) * |
| <18.5 | 1,863 (7.2) |
| 18.5–24.9 | 17,362 (66.7) |
| 25.0–29.9 | 4,879 (18.7) |
| ≥30.0 | 522 (2.0) |
| Missing | 1,411 (5.4) |
| Musculoskeletal disease |  |
| Yes | 2,912 (11.2) |
| No | 21,341 (82.0) |
| Missing | 1,784 (6.9) |
| Depression |  |
| None (GDS<5) | 15,592 (59.9) |
| Mild (GDS of 5–9) | 4,178 (16.0) |
| Severe (GDS≥10) | 1,485 (5.7) |
| Missing | 4,782 (18.4) |
| Physical activity |  |
| ≥4 times a week | 7,237 (27.7) |
| 2–3 times a week | 4,695 (18.0) |
| Once a week | 2,230 (8.6) |
| 1–3 times a month | 2,123 (8.2) |
| A few times a year | 1,530 (5.9) |
| Rare | 5,801 (22.3) |
| Missing | 2,421 (9.3) |
| Smoking |  |
| Current | 2,584 (9.9) |
| Former | 3,985 (15.3) |
| Never | 18,971 (72.9) |
| Missing | 497 (1.9) |
| Drinking habit |  |
| Current | 8,855 (34.0) |
| Former | 1,331 (5.1) |
| Never | 15,377 (59.1) |
| Missing | 474 (1.8) |

Note: * mean BMI (SD).

**Table S3.** The associations of each parameter with low back pain in complete data (n = 24,285. Multilevel Poisson regression analysis).

| Parameters  (Fixed parameters) | Model 1 | | Model 2 | | Model 3 | | Model 4 | |
| --- | --- | --- | --- | --- | --- | --- | --- | --- |
|  | PR | 95% CI | PR | 95% CI | PR | 95% CI | PR | 95% CI |
| Education, years (ref, ≥13) | | | | | | | | |
| 10–12 | 1.05 | 1.01, 1.10 | 1.04 | 0.99, 1.09 | 1.04 | 0.99, 1.09 | 1.02 | 0.96, 1.07 |
| <10 | 1.12 | 1.07, 1.17 | 1.09 | 1.04, 1.13 | 1.07 | 1.02, 1.13 | 1.04 | 0.98, 1.10 |
| Sex (ref, male) |  | |  | |  | |  | |
| Female | - | | 1.07 | 1.03, 1.10 | 1.06 | 1.01, 1.10 | 1.07 | 1.01, 1.13 |
| Age (ref, 65–69) |  | |  | |  | |  | |
| 70–74 | - | | 1.00 | 0.96, 1.05 | 1.01 | 0.96, 1.05 | 1.01 | 0.96, 1.07 |
| 75–79 | - | | 1.06 | 1.01, 1.10 | 1.03 | 0.98, 1.09 | 1.03 | 0.97, 1.10 |
| 80–84 | - | | 1.12 | 1.06, 1.18 | 1.08 | 1.01, 1.16 | 1.10 | 1.02, 1.18 |
| ≥85 |  | | 1.13 | 1.05, 1.20 | 1.09 | 0.99, 1.19 | 1.10 | 0.996, 1.22 |
| Number of persons living together (ref, living with others) | | | | | | | | |
| Alone | - | | - | | 1.00 | 0.93, 1.07 | 0.97 | 0.90, 1.05 |
| Marital status (ref, married) | | | | | | | | |
| Widowed | - | | - | | 0.99 | 0.94, 1.05 | 0.98 | 0.91, 1.04 |
| Divorced | - | | - | | 1.03 | 0.92, 1.16 | 1.01 | 0.89, 1.12 |
| Never married | - | | - | | 0.99 | 0.87, 1.12 | 0.97 | 0.82, 1.11 |
| Musculoskeletal disease (ref, no) | | | | | | | | |
| Yes | - | | - | | 1.34 | 1.27, 1.40 | 1.31 | 1.24, 1.39 |
| Physical activity (ref, ≥4 times a week) | | | | | | | | |
| 2–3 times a week | - | | - | | 0.96 | 0.90, 1.03 | 0.97 | 0.91, 1.05 |
| Once a week | - | | - | | 1.01 | 0.94, 1.08 | 0.99 | 0.92, 1.07 |
| 1–3 times a month | - | | - | | 1.02 | 0.94, 1.11 | 1.03 | 0.94, 1.13 |
| A few times a year | - | | - | | 1.07 | 0.97, 1.17 | 1.05 | 0.96, 1.17 |
| Rare | - | | - | | 1.06 | 0.98, 1.13 | 1.02 | 0.95, 1.10 |
| BMI (ref, 18.5–24.9) |  | |  | |  | |  | |
| <18.5 | - | | - | | 0.98 | 0.91, 1.05 | 0.97 | 0.90, 1.05 |
| 25.0–29.9 |  | |  | | 1.07 | 1.02, 1.12 | 1.08 | 1.03, 1.13 |
| ≥30.0 | - | | - | | 1.15 | 1.02, 1.29 | 1.14 | 1.01, 1.30 |
| Smoking (ref, never) |  | |  | |  | |  | |
| Former | - | | - | | 1.06 | 0.99, 1.12 | 1.04 | 0.98, 1.11 |
| Current | - | | - | | 1.01 | 0.95, 1.08 | 1.01 | 0.94, 1.08 |
| Drinking habit (ref, never) |  | |  | |  | |  | |
| Former | - | | - | | 1.05 | 0.96, 1.15 | 1.05 | 0.95, 1.16 |
| Current | - | | - | | 1.04 | 0.99, 1.09 | 1.05 | 1.003, 1.11 |
| Depression (ref, non-depression) | | | | | | | | |
| Mild depression | - | | - | | - | | 1.20 | 1.14, 1.26 |
| Severe depression | - | | - | | - | | 1.31 | 1.21, 1.41 |
| Past occupation (ref, professionals) | | | | | | | | |
| White-collared workers | 1.02 | 0.96, 1.07 | 1.00 | 0.95, 1.06 | 1.01 | 0.95, 1.07 | 1.01 | 0.95, 1.08 |
| Blue-collared workers | 1.08 | 1.03, 1.14 | 1.07 | 1.02, 1.12 | 1.07 | 1.01, 1.13 | 1.05 | 0.99, 1.11 |
| Never worked before | 1.11 | 1.02, 1.20 | 1.03 | 0.95, 1.13 | 1.00 | 0.90, 1.10 | 0.99 | 0.89, 1.11 |
| Sex (ref, male) |  | |  | |  | |  | |
| Female | - | | 1.07 | 1.03, 1.11 | 1.06 | 1.01, 1.12 | 1.08 | 1.02, 1.15 |
| Age (ref, 65–69) |  | |  | |  | |  | |
| 70–74 | - | | 1.01 | 0.96, 1.06 | 1.01 | 0.96, 1.06 | 1.01 | 0.96, 1.07 |
| 75–79 | - | | 1.06 | 1.004, 1.11 | 1.03 | 0.97, 1.09 | 1.03 | 0.96, 1.09 |
| 80–84 |  | | 1.12 | 1.06, 1.19 | 1.09 | 1.02, 1.17 | 1.10 | 1.03, 1.19 |
| ≥85 | - | | 1.13 | 1.04, 1.22 | 1.09 | 0.99, 1.20 | 1.10 | 0.99, 1.22 |
| Number of persons living together (ref, living with others) | | | | | | | | |
| Alone | - | | - | | 0.99 | 0.92, 1.07 | 0.96 | 0.88, 1.04 |
| Marital status (ref, married) | | | | | | | | |
| Widowed | - | | - | | 1.00 | 0.94, 1.07 | 0.99 | 0.92, 1.06 |
| Divorced | - | | - | | 1.04 | 0.92, 1.18 | 1.02 | 0.90, 1.16 |
| Never married | - | | - | | 0.96 | 0.83, 1.11 | 0.95 | 0.81, 1.09 |
| Musculoskeletal disease (ref, no) | | | | | | | | |
| Yes | - | | - | | 1.33 | 1.26, 1.41 | 1.31 | 1.23, 1.39 |
| Physical activity (ref, ≥4 times a week) | | | | | | | | |
| 2–3 times a week | - | | - | | 0.97 | 0.90, 1.05 | 0.98 | 0.90, 1.06 |
| Once a week | - | | - | | 1.01 | 0.93, 1.09 | 0.99 | 0.91, 1.08 |
| 1–3 times a month | - | | - | | 1.04 | 0.95, 1.14 | 1.05 | 0.94, 1.15 |
| A few times a year | - | | - | | 1.06 | 0.96, 1.17 | 1.05 | 0.94, 1.17 |
| Rare | - | | - | | 1.07 | 0.99, 1.15 | 1.03 | 0.94, 1.12 |
| BMI (ref, 18.5–24.9) |  | |  | |  | |  | |
| <18.5 | - | | - | | 0.99 | 0.91, 1.07 | 0.97 | 0.89, 1.06 |
| 25.0–29.9 |  | |  | | 1.06 | 1.02, 1.12 | 1.07 | 1.01, 1.13 |
| ≥30.0 | - | | - | | 1.15 | 1.01, 1.31 | 1.13 | 0.98, 1.30 |
| Smoking (ref, never) | | | | | | | | |
| Current | - | | - | | 1.04 | 0.98, 1.11 | 1.03 | 0.96, 1.10 |
| Former | - | | - | | 1.01 | 0.93, 1.08 | 1.00 | 0.93, 1.08 |
| Drinking habit (ref, never) | | | | | | | | |
| Current | - | | - | | 1.06 | 0.96, 1.16 | 1.07 | 0.96, 1.17 |
| Former | - | | - | | 1.04 | 0.99, 1.09 | 1.07 | 1.01, 1.12 |
| Depression (ref, non-depression) | | | | | | | | |
| Mild depression | - | | - | | - | | 1.19 | 1.13, 1.26 |
| Severe depression | - | | - | | - | | 1.31 | 1.20, 1.42 |
| Income, yen (ref, ≥3 million) | | | | | | | | |
| 2 million–2.99 million | 1.03 | 0.97, 1.08 | 1.02 | 0.97, 1.08 | 1.03 | 0.97, 1.09 | 1.01 | 0.95, 1.07 |
| 1 million–1.99 million | 1.09 | 1.04, 1.14 | 1.09 | 1.04, 1.14 | 1.08 | 1.02, 1.13 | 1.05 | 0.99, 1.10 |
| <1 million | 1.20 | 1.14, 1.27 | 1.18 | 1.11, 1.25 | 1.17 | 1.09, 1.25 | 1.12 | 1.04, 1.20 |
| Sex (ref, male) |  | |  | |  | |  | |
| Female | - | | 1.06 | 1.02, 1.09 | 1.05 | 0.99, 1.10 | 1.06 | 0.998, 1.12 |
| Age (ref, 65–69) |  | |  | |  | |  | |
| 70–74 | - | | 1.00 | 0.96, 1.05 | 1.00 | 0.95, 1.06 | 1.01 | 0.95, 1.06 |
| 75–79 | - | | 1.05 | 1.001, 1.11 | 1.03 | 0.97, 1.09 | 1.02 | 0.96, 1.09 |
| 80–84 |  | | 1.12 | 1.05, 1.18 | 1.09 | 1.02, 1.17 | 1.09 | 1.01, 1.17 |
| ≥85 | - | | 1.14 | 1.06, 1.24 | 1.08 | 0.98, 1.19 | 1.09 | 0.98, 1.21 |
| Number of persons living together (ref, living with others) | | | | | | | | |
| Alone | - | | - | | 0.97 | 0.91, 1.05 | 0.96 | 0.89, 1.04 |
| Marital status (ref, married) | | | | | | | | |
| Widowed | - | | - | | 1.00 | 0.94, 1.07 | 0.98 | 0.91, 1.04 |
| Divorced | - | | - | | 1.03 | 0.91, 1.16 | 1.01 | 0.89, 1.14 |
| Never married | - | | - | | 0.98 | 0.84, 1.12 | 0.96 | 0.82, 1.10 |
| Musculoskeletal disease (ref, no) | | | | | | | | |
| Yes | - | | - | | 1.33 | 1.25, 1.41 | 1.31 | 1.22, 1.39 |
| Physical activity (ref, ≥4 times a week) | | | | | | | | |
| 2–3 times a week | - | | - | | 0.96 | 0.89, 1.03 | 0.97 | 0.90, 1.05 |
| Once a week | - | | - | | 0.99 | 0.92, 1.07 | 0.98 | 0.90, 1.07 |
| 1–3 times a month | - | | - | | 1.02 | 0.94, 1.12 | 1.03 | 0.93, 1.13 |
| A few times a year | - | | - | | 1.07 | 0.96, 1.18 | 1.05 | 0.94, 1.16 |
| Rare | - | | - | | 1.06 | 0.98, 1.14 | 1.03 | 0.95, 1.11 |
| BMI (ref, 18.5–24.9) |  | |  | |  | |  | |
| <18.5 | - | | - | | 0.97 | 0.89, 1.05 | 0.95 | 0.87, 1.04 |
| 25.0–29.9 |  | |  | | 1.07 | 1.02, 1.12 | 1.08 | 1.02, 1.14 |
| ≥30.0 | - | | - | | 1.16 | 1.02, 1.32 | 1.15 | 1.004, 1.30 |
| Smoking (ref, never) | | | | | | | | |
| Current | - | | - | | 1.05 | 0.99, 1.11 | 1.04 | 0.97, 1.11 |
| Former | - | | - | | 1.02 | 0.95, 1.09 | 1.00 | 0.93, 1.08 |
| Drinking habit (ref, never) | | | | | | | | |
| Current | - | | - | | 1.06 | 0.97, 1.16 | 1.06 | 0.95, 1.16 |
| Former | - | | - | | 1.03 | 0.99, 1.08 | 1.05 | 0.997, 1.10 |
| Depression (ref, non-depression) | | | | | | | | |
| Mild depression | - | | - | | - | | 1.19 | 1.13, 1.26 |
| Severe depression | - | | - | | - | | 1.29 | 1.20, 1.39 |
| Subjective economic situation (ref, very comfortable) | | | | | | | | |
| Comfortable | 1.06 | 1.00, 1.13 | 1.07 | 1.005, 1.13 | 1.05 | 0.98, 1.13 | 1.04 | 0.96, 1.11 |
| Difficult | 1.19 | 1.12, 1.27 | 1.22 | 1.14, 1.29 | 1.18 | 1.10, 1.27 | 1.13 | 1.04, 1.21 |
| Very difficult | 1.34 | 1.24, 1.45 | 1.37 | 1.27, 1.48 | 1.32 | 1.20, 1.45 | 1.16 | 1.05, 1.28 |
| Sex (ref, male) |  | |  | |  | |  | |
| Female | - | | 1.08 | 1.05, 1.11 | 1.07 | 1.02, 1.12 | 1.08 | 1.02, 1.13 |
| Age (ref, 65–69) |  | |  | |  | |  | |
| 70–74 | - | | 1.01 | 0.97, 1.05 | 1.01 | 0.96, 1.06 | 1.02 | 0.97, 1.07 |
| 75–79 | - | | 1.07 | 1.02, 1.12 | 1.04 | 0.99, 1.10 | 1.04 | 0.98, 1.10 |
| 80–84 |  | | 1.16 | 1.10, 1.22 | 1.12 | 1.05, 1.19 | 1.12 | 1.05, 1.20 |
| ≥85 | - | | 1.18 | 1.10, 1.27 | 1.14 | 1.05, 1.24 | 1.13 | 1.02, 1.25 |
| Number of persons living together (ref, living with others) | | | | | | | | |
| Alone | - | | - | | 0.99 | 0.92, 1.05 | 0.96 | 0.90, 1.04 |
| Marital status (ref, married) | | | | | | | | |
| Widowed | - | | - | | 0.99 | 0.93, 1.05 | 0.98 | 0.92, 1.04 |
| Divorced | - | | - | | 0.99 | 0.88, 1.10 | 1.00 | 0.89, 1.12 |
| Never married | - | | - | | 0.98 | 0.86, 1.11 | 0.96 | 0.83, 1.10 |
| Musculoskeletal disease (ref, no) | | | | | | | | |
| Yes | - | | - | | 1.32 | 1.25, 1.39 | 1.30 | 1.23, 1.38 |
| Physical activity (ref, ≥4 times a week) | | | | | | | | |
| 2–3 times a week | - | | - | | 0.97 | 0.90, 1.04 | 0.98 | 0.91, 1.06 |
| Once a week | - | | - | | 1.00 | 0.93, 1.08 | 0.99 | 0.92, 1.07 |
| 1–3 times a month | - | | - | | 1.02 | 0.93, 1.11 | 1.03 | 0.94, 1.13 |
| A few times a year | - | | - | | 1.06 | 0.97, 1.16 | 1.05 | 0.95, 1.16 |
| Rare | - | | - | | 1.04 | 0.97, 1.12 | 1.02 | 0.94, 1.10 |
| BMI (ref, 18.5–24.9) |  | |  | |  | |  | |
| <18.5 | - | | - | | 0.98 | 0.91, 1.05 | 0.97 | 0.89, 1.05 |
| 25.0–29.9 |  | |  | | 1.07 | 1.02, 1.12 | 1.08 | 1.03, 1.13 |
| ≥30.0 | - | | - | | 1.15 | 1.01, 1.29 | 1.14 | 1.004, 1.29 |
| Smoking (ref, never) | | | | | | | | |
| Current | - | | - | | 1.05 | 0.99, 1.11 | 1.04 | 0.97, 1.10 |
| Former | - | | - | | 1.01 | 0.94, 1.08 | 1.01 | 0.94, 1.08 |
| Drinking habit (ref, never) | | | | | | | | |
| Current | - | | - | | 1.05 | 0.96, 1.15 | 1.06 | 0.96, 1.16 |
| Former | - | | - | | 1.04 | 0.99, 1.08 | 1.05 | 1.003, 1.10 |
| Depression (ref, non-depression) | | | | | | | | |
| Mild depression | - | | - | | - | | 1.16 | 1.10, 1.22 |
| Severe depression | - | | - | | - | | 1.25 | 1.15, 1.34 |
| Wealth, yen (ref, ≥50 million) | | | | | | | | |
| 10 million–49.99 million | 1.07 | 1.01, 1.13 | 1.07 | 1.01, 1.13 | 1.05 | 0.99, 1.12 | 1.04 | 0.97, 1.11 |
| 5 million–9.99 million | 1.08 | 1.01, 1.15 | 1.08 | 1.01, 1.16 | 1.07 | 0.996, 1.16 | 1.05 | 0.97, 1.13 |
| 1 million–4.99 million | 1.15 | 1.08, 1.23 | 1.14 | 1.07, 1.22 | 1.11 | 1.03, 1.20 | 1.07 | 0.99, 1.16 |
| <1 million | 1.23 | 1.14, 1.32 | 1.22 | 1.14, 1.31 | 1.19 | 1.09, 1.29 | 1.13 | 1.03, 1.24 |
| Sex (ref, male) |  | |  | |  | |  | |
| Female | - | | 1.06 | 1.02, 1.10 | 1.05 | 0.998, 1.11 | 1.06 | 1.003, 1.12 |
| Age (ref, 65–69) |  | |  | |  | |  | |
| 70–74 | - | | 1.01 | 0.97, 1.06 | 1.01 | 0.96, 1.07 | 1.01 | 0.96, 1.07 |
| 75–79 | - | | 1.06 | 1.01, 1.12 | 1.04 | 0.98, 1.11 | 1.03 | 0.97, 1.10 |
| 80–84 |  | | 1.14 | 1.07, 1.21 | 1.10 | 1.02, 1.18 | 1.10 | 1.01, 1.19 |
| ≥85 | - | | 1.16 | 1.06, 1.25 | 1.11 | 1.01, 1.23 | 1.10 | 0.99, 1.22 |
| Number of persons living together (ref, living with others) | | | | | | | | |
| Alone | - | | - | | 0.97 | 0.90, 1.05 | 0.96 | 0.88, 1.04 |
| Marital status (ref, married) | | | | | | | | |
| Widowed | - | | - | | 1.00 | 0.93, 1.07 | 0.98 | 0.91, 1.05 |
| Divorced | - | | - | | 1.01 | 0.89, 1.14 | 1.01 | 0.87, 1.15 |
| Never married | - | | - | | 0.96 | 0.82, 1.12 | 0.94 | 0.80, 1.09 |
| Musculoskeletal disease (ref, no) | | | | | | | | |
| Yes | - | | - | | 1.33 | 1.25, 1.42 | 1.31 | 1.22, 1.40 |
| Physical activity (ref, ≥4 times a week) | | | | | | | | |
| 2–3 times a week | - | | - | | 0.96 | 0.89, 1.03 | 0.97 | 0.90, 1.05 |
| Once a week | - | | - | | 0.99 | 0.91, 1.07 | 0.98 | 0.90, 1.06 |
| 1–3 times a month | - | | - | | 1.03 | 0.94, 1.12 | 1.03 | 0.94, 1.14 |
| A few times a year | - | | - | | 1.05 | 0.95, 1.16 | 1.04 | 0.93, 1.15 |
| Rare | - | | - | | 1.04 | 0.96, 1.12 | 1.01 | 0.94, 1.10 |
| BMI (ref, 18.5–24.9) |  | |  | |  | |  | |
| <18.5 | - | | - | | 0.96 | 0.88, 1.04 | 0.95 | 0.87, 1.04 |
| 25.0–29.9 |  | |  | | 1.07 | 1.02, 1.13 | 1.08 | 1.02, 1.14 |
| ≥30.0 | - | | - | | 1.16 | 1.01, 1.32 | 1.15 | 0.99, 1.33 |
| Smoking (ref, never) |  | |  | |  | |  | |
| Current | - | | - | | 1.06 | 0.99, 1.13 | 1.04 | 0.97, 1.11 |
| Former | - | | - | | 1.01 | 0.94, 1.09 | 1.00 | 0.92, 1.08 |
| Drinking habit (ref, never) | | | | | | | | |
| Current | - | | - | | 1.04 | 0.94, 1.14 | 1.03 | 0.94, 1.14 |
| Former | - | | - | | 1.03 | 0.98, 1.08 | 1.04 | 0.99, 1.10 |
| Depression (ref, non-depression) | | | | | | | | |
| Mild depression | - | | - | | - | | 1.19 | 1.13, 1.26 |
| Severe depression | - | | - | | - | | 1.29 | 1.18, 1.41 |

Abbreviations: PR, prevalence ratio; 95 % CI, 95% credible interval.

Socioeconomic status was separately added to each model. Model 1, a crude model; Model 2, with age and sex adjusted to Model 1; Model 3, with number of persons living together, marital status, musculoskeletal disease, BMI, drinking habit, smoking and physical activity added to Model 2; Model 4, with depression added to Model 3.

**Table S4.** The association of socioeconomic status with low back pain, stratified by sex or age after multiple data imputations (n = 26,037. Multilevel Poisson regression analysis).

| Socioeconomic status  (Fixed parameters) | Model 1-a | | Model 2-a | | | Model 3-a | | | Model 4-a | | |
| --- | --- | --- | --- | --- | --- | --- | --- | --- | --- | --- | --- |
|  | PR | 95% CI | PR | 95% CI | PR | | | 95% CI | PR | 95% CI | |
| Male |  | |  | | |  | | |  | | |
| Education, years (ref, ≥13) | | | | | | | | | | | |
| 10–12 | 1.04 | 0.98, 1.11 | 1.04 | 0.98, 1.10 | 1.04 | | 0.97, 1.11 | | 1.01 | | 0.94, 1.09 |
| <10 | 1.10 | 1.03, 1.17 | 1.09 | 1.02, 1.16 | 1.08 | | 1.004, 1.16 | | 1.04 | | 0.96, 1.12 |
| Past occupation (ref, professionals) | | | | | | | | | | | |
| White-collared workers | 0.99 | 0.92, 1.06 | 0.98 | 0.91, 1.06 | 0.98 | | 0.91, 1.06 | | 0.98 | | 0.90, 1.07 |
| Blue-collared workers | 1.08 | 1.01, 1.15 | 1.07 | 1.01, 1.14 | 1.07 | | 1.002, 1.14 | | 1.05 | | 0.99, 1.13 |
| Never worked before | 1.12 | 0.77, 1.54 | 1.10 | 0.76, 1.51 | 0.95 | | 0.56, 1.45 | | 1.01 | | 0.58, 1.55 |
| Income, yen (ref, ≥3 million) | | | | | | | | | | | |
| 2 million–2.99 million | 1.04 | 0.96, 1.11 | 1.04 | 0.96, 1.11 | 1.04 | | 0.96, 1.13 | | 1.02 | | 0.94, 1.10 |
| 1 million–1.99 million | 1.11 | 1.04, 1.19 | 1.11 | 1.04, 1.18 | 1.10 | | 1.02, 1.18 | | 1.06 | | 0.99, 1.14 |
| <1 million | 1.19 | 1.09, 1.30 | 1.18 | 1.08, 1.29 | 1.18 | | 1.07, 1.31 | | 1.12 | | 0.99, 1.26 |
| Subjective economic situation (ref, very comfortable) | | | | | | | | | | | |
| Comfortable | 1.08 | 0.98, 1.19 | 1.09 | 0.99, 1.19 | 1.07 | | 0.96, 1.18 | | 1.05 | | 0.94, 1.17 |
| Difficult | 1.23 | 1.13, 1.35 | 1.25 | 1.13, 1.38 | 1.22 | | 1.10, 1.35 | | 1.16 | | 1.04, 1.30 |
| Very difficult | 1.40 | 1.25, 1.57 | 1.41 | 1.25, 1.59 | 1.38 | | 1.21, 1.57 | | 1.21 | | 1.04, 1.41 |
| Wealth, yen (ref, ≥50 million) | | | | | | | | | | | |
| 10 million–49.99 million | 1.06 | 0.98, 1.14 | 1.06 | 0.98, 1.15 | 1.05 | | 0.96, 1.14 | | 1.03 | | 0.94, 1.12 |
| 5 million–9.99 million | 1.05 | 0.96, 1.15 | 1.05 | 0.95, 1.15 | 1.04 | | 0.94, 1.15 | | 0.995 | | 0.90, 1.10 |
| 1 million–4.99 million | 1.14 | 1.04, 1.26 | 1.14 | 1.03, 1.25 | 1.14 | | 1.03, 1.27 | | 1.09 | | 0.98, 1.22 |
| <1 million | 1.18 | 1.06, 1.31 | 1.18 | 1.06, 1.30 | 1.15 | | 1.02, 1.29 | | 1.08 | | 0.94, 1.22 |
| Female |  | |  | | |  | | |  | | |
| Education, years (ref, ≥13) | | | | | | | | | | | |
| 10–12 | 1.05 | 0.99, 1.13 | 1.04 | 0.98, 1.12 | 1.03 | | 0.95, 1.11 | | 1.02 | | 0.94, 1.11 |
| <10 | 1.11 | 1.04, 1.19 | 1.08 | 1.01, 1.16 | 1.07 | | 0.98, 1.15 | | 1.04 | | 0.95, 1.13 |
| Past occupation (ref, professionals) | | | | | | | | | | | |
| White-collared workers | 1.02 | 0.94, 1.11 | 1.02 | 0.93, 1.11 | 1.04 | | 0.95, 1.15 | | 1.06 | | 0.95, 1.18 |
| Blue-collared workers | 1.07 | 0.99, 1.16 | 1.07 | 0.98, 1.16 | 1.07 | | 0.98, 1.18 | | 1.06 | | 0.95, 1.18 |
| Never worked before | 1.07 | 0.97, 1.18 | 1.02 | 0.92, 1.14 | 1.01 | | 0.90, 1.13 | | 1.004 | | 0.88, 1.14 |
| Income, yen (ref, ≥3 million) | | | | | | | | | | | |
| 2 million–2.99 million | 1.02 | 0.95, 1.10 | 1.02 | 0.95, 1.09 | 1.01 | | 0.94, 1.10 | | 0.999 | | 0.92, 1.09 |
| 1 million–1.99 million | 1.07 | 0.99, 1.14 | 1.06 | 0.99, 1.13 | 1.05 | | 0.97, 1.13 | | 1.03 | | 0.94, 1.11 |
| <1 million | 1.19 | 1.11, 1.28 | 1.17 | 1.08, 1.26 | 1.15 | | 1.05, 1.25 | | 1.11 | | 1.01, 1.22 |
| Subjective economic situation (ref, very comfortable) | | | | | | | | | | | |
| Comfortable | 1.05 | 0.97, 1.14 | 1.06 | 0.98, 1.15 | 1.04 | | 0.95, 1.15 | | 1.03 | | 0.93, 1.13 |
| Difficult | 1.17 | 1.08, 1.27 | 1.20 | 1.10, 1.30 | 1.16 | | 1.05, 1.27 | | 1.10 | | 0.99, 1.23 |
| Very difficult | 1.32 | 1.18, 1.45 | 1.34 | 1.22, 1.49 | 1.28 | | 1.12, 1.45 | | 1.12 | | 0.96, 1.29 |
| Wealth, yen (ref, ≥50 million) | | | | | | | | | | | |
| 10 million–49.99 million | 1.08 | 0.99, 1.17 | 1.08 | 0.99, 1.17 | 1.05 | | 0.96, 1.15 | | 1.05 | | 0.95, 1.15 |
| 5 million–9.99 million | 1.12 | 1.02, 1.23 | 1.12 | 1.01, 1.22 | 1.11 | | 0.99, 1.23 | | 1.10 | | 0.97, 1.23 |
| 1 million–4.99 million | 1.16 | 1.05, 1.28 | 1.14 | 1.03, 1.26 | 1.09 | | 0.97, 1.21 | | 1.04 | | 0.92, 1.18 |
| <1 million | 1.27 | 1.15, 1.41 | 1.25 | 1.12, 1.38 | 1.21 | | 1.07, 1.37 | | 1.19 | | 1.04, 1.35 |
| Socioeconomic status | Model 1-b | | Model 2-b | | | Model 3-b | | | Model 4-b | | |
|  | PR | 95% CI | PR | 95% CI | PR | | 95% CI | | PR | | 95% CI |
| Age≥75 |  | |  | | |  | | |  | | |
| Education, years (ref, ≥13) | | | | | | | | | | | |
| 10–12 | 1.07 | 0.99, 1.17 | 1.05 | 0.97, 1.14 | 1.03 | | 0.94, 1.12 | | 0.99 | | 0.90, 1.09 |
| <10 | 1.13 | 1.06, 1.22 | 1.11 | 1.03, 1.19 | 1.08 | | 1.0001, 1.17 | | 1.03 | | 0.98, 1.13 |
| Past occupation (ref, professionals) | | | | | | | | | | | |
| White-collared workers | 1.02 | 0.94, 1.12 | 1.01 | 0.92, 1.10 | 1.02 | | 0.92, 1.12 | | 1.02 | | 0.91, 1.14 |
| Blue-collared workers | 1.10 | 1.01, 1.19 | 1.09 | 1.00, 1.18 | 1.08 | | 0.98, 1.18 | | 1.06 | | 0.96, 1.17 |
| Never worked before | 1.14 | 1.02, 1.27 | 1.08 | 0.96, 1.20 | 1.05 | | 0.91, 1.21 | | 1.03 | | 0.88, 1.19 |
| Income, yen (ref, ≥3 million) | | | | | | | | | | | |
| 2 million–2.99 million | 1.05 | 0.97, 1.14 | 1.05 | 0.96, 1.14 | 1.06 | | 0.96, 1.16 | | 1.06 | | 0.95, 1.17 |
| 1 million–1.99 million | 1.09 | 1.01, 1.17 | 1.09 | 1.01, 1.18 | 1.07 | | 0.97, 1.17 | | 1.04 | | 0.94, 1.14 |
| <1 million | 1.24 | 1.13, 1.34 | 1.21 | 1.11, 1.32 | 1.21 | | 1.08, 1.34 | | 1.17 | | 1.05, 1.31 |
| Subjective economic situation (ref, very comfortable) | | | | | | | | | | | |
| Comfortable | 1.06 | 0.98, 1.16 | 1.06 | 0.98, 1.16 | 1.07 | | 0.96, 1.19 | | 1.05 | | 0.94, 1.17 |
| Difficult | 1.20 | 1.10, 1.32 | 1.21 | 1.11, 1.32 | 1.20 | | 1.08, 1.34 | | 1.14 | | 1.02, 1.28 |
| Very difficult | 1.33 | 1.18, 1.49 | 1.33 | 1.19, 1.48 | 1.34 | | 1.16, 1.54 | | 1.18 | | 0.998, 1.40 |
| Wealth, yen (ref, ≥50 million) | | | | | | | | | | | |
| 10 million–49.99 million | 1.08 | 0.99, 1.18 | 1.07 | 0.98, 1.18 | 1.06 | | 0.95, 1.17 | | 1.04 | | 0.93, 1.16 |
| 5 million–9.99 million | 1.08 | 0.98, 1.20 | 1.07 | 0.96, 1.19 | 1.07 | | 0.95, 1.20 | | 1.03 | | 0.90, 1.18 |
| 1 million–4.99 million | 1.14 | 1.03, 1.27 | 1.13 | 1.01, 1.26 | 1.11 | | 0.98, 1.25 | | 1.07 | | 0.93, 1.21 |
| <1 million | 1.27 | 1.13, 1.41 | 1.25 | 1.12, 1.39 | 1.22 | | 1.07, 1.39 | | 1.16 | | 0.998, 1.34 |
| Age<75 |  | |  | | |  | | |  | | |
| Education, years (ref, ≥13) | | | | | | | | | | | |
| 10–12 | 1.04 | 0.98, 1.10 | 1.03 | 0.98, 1.10 | 1.04 | | 0.98, 1.10 | | 1.03 | | 0.96, 1.10 |
| <10 | 1.08 | 1.02, 1.14 | 1.08 | 1.01, 1.14 | 1.07 | | 1.001, 1.14 | | 1.04 | | 0.98, 1.11 |
| Past occupation (ref, professionals) | | | | | | | | | | | |
| White-collared workers | 1.01 | 0.95, 1.08 | 1.00 | 0.93, 1.07 | 1.01 | | 0.93, 1.08 | | 1.02 | | 0.93, 1.10 |
| Blue-collared workers | 1.07 | 1.01, 1.14 | 1.06 | 1.00, 1.13 | 1.06 | | 0.99, 1.14 | | 1.04 | | 0.97, 1.13 |
| Never worked before | 1.00 | 0.88, 1.14 | 0.97 | 0.85, 1.10 | 0.92 | | 0.79, 1.07 | | 0.94 | | 0.79, 1.11 |
| Income, yen (ref, ≥3 million) | | | | | | | | | | | |
| 2 million–2.99 million | 1.01 | 0.95, 1.08 | 1.01 | 0.94, 1.08 | 1.01 | | 0.94, 1.08 | | 0.98 | | 0.91, 1.06 |
| 1 million–1.99 million | 1.08 | 1.01, 1.14 | 1.08 | 1.02, 1.15 | 1.08 | | 1.01, 1.15 | | 1.06 | | 0.99, 1.13 |
| <1 million | 1.16 | 1.07, 1.25 | 1.15 | 1.07, 1.24 | 1.14 | | 1.04, 1.25 | | 1.08 | | 0.97, 1.19 |
| Subjective economic situation (ref, very comfortable) | | | | | | | | | | | |
| Comfortable | 1.07 | 0.98, 1.17 | 1.07 | 0.98, 1.16 | 1.03 | | 0.94, 1.14 | | 1.02 | | 0.92, 1.13 |
| Difficult | 1.20 | 1.10, 1.31 | 1.21 | 1.10, 1.32 | 1.16 | | 1.05, 1.28 | | 1.10 | | 0.99, 1.22 |
| Very difficult | 1.38 | 1.24, 1.53 | 1.38 | 1.24, 1.53 | 1.30 | | 1.15, 1.47 | | 1.13 | | 0.98, 1.29 |
| Wealth, yen (ref, ≥50 million) | | | | | | | | | | | |
| 10 million–49.99 million | 1.06 | 0.99, 1.14 | 1.07 | 0.99, 1.15 | 1.05 | | 0.97, 1.13 | | 1.04 | | 0.96, 1.13 |
| 5 million–9.99 million | 1.09 | 1.00, 1.18 | 1.09 | 1.00, 1.19 | 1.08 | | 0.99, 1.18 | | 1.06 | | 0.96, 1.17 |
| 1 million–4.99 million | 1.15 | 1.06, 1.25 | 1.15 | 1.05, 1.25 | 1.12 | | 1.02, 1.23 | | 1.07 | | 0.97, 1.20 |
| <1 million | 1.19 | 1.08, 1.31 | 1.19 | 1.08, 1.32 | 1.17 | | 1.05, 1.31 | | 1.12 | | 0.99, 1.25 |

Abbreviations: PR, prevalence ratio; 95 % CI, 95% credible interval.

Socioeconomic status was separately added to each model.

Model 1-a, a crude model; Model 2-a, with age adjusted to Model 1; Model 3-a, with number of persons living together, marital status, musculoskeletal disease, BMI, drinking habit, smoking and physical activity added to Model 2; Model 4-a, with depression added to Model 3-a.

Model 1-b, a crude model; Model 2-a, with sex adjusted to Model 1; Model 3-b, with number of persons living together, marital status, musculoskeletal disease, BMI, drinking habit, smoking and physical activity added to Model 2; Model 4-b, with depression added to Model 3-b.

**Table S5.** The association of socioeconomic status with *severe* low back pain, stratified by sex or age in the complete dataset (n = 16,762. Separately Multilevel Poisson regression analysis).

| Socioeconomic status  (Fixed parameters) | Model 1-a | | Model 2-a | | Model 3-a | | | Model 4-a | |
| --- | --- | --- | --- | --- | --- | --- | --- | --- | --- |
|  | PR | 95% CI | PR | 95% CI | PR | | 95% CI | PR | 95% CI |
| Male |  | |  | |  | | |  | |
| Education, years (ref, ≥13) | | | | | | | | | |
| 10–12 | 1.10 | 1.00, 1.20 | 1.09 | 0.99, 1.20 | 1.09 | 0.98, 1.20 | | 1.03 | 0.92, 1.15 |
| <10 | 1.29 | 1.18, 1.41 | 1.24 | 1.14, 1.36 | 1.18 | 1.06, 1.31 | | 1.11 | 0.99, 1.23 |
| Past occupation (ref, professionals) | | | | | | | | | |
| White-collared workers | 0.92 | 0.85, 1.05 | 0.91 | 0.81, 1.01 | 0.93 | 0.82, 1.04 | | 0.93 | 0.81, 1.05 |
| Blue-collared workers | 1.14 | 1.06, 1.26 | 1.13 | 1.03, 1.23 | 1.10 | 1.001, 1.22 | | 1.05 | 0.94, 1.17 |
| Never worked before | 1.33 | 1.00, 1.83 | 1.21 | 0.71, 1.82 | 0.88 | 0.38, 1.54 | | 0.82 | 0.37, 1.51 |
| Income, yen (ref, ≥3 million) | | | | | | | | | |
| 2 million–2.99 million | 1.09 | 0.97, 1.22 | 1.08 | 0.97, 1.20 | 1.07 | 0.94, 1.20 | | 1.04 | 0.92, 1.18 |
| 1 million–1.99 million | 1.26 | 1.16, 1.39 | 1.26 | 1.15, 1.39 | 1.22 | 1.09, 1.35 | | 1.13 | 1.003, 1.27 |
| <1 million | 1.57 | 1.39, 1.76 | 1.54 | 1.36, 1.73 | 1.47 | 1.27, 1.68 | | 1.28 | 1.08, 1.50 |
| Subjective economic situation (ref, very comfortable) | | | | | | | | | |
| Comfortable | 1.17 | 1.01, 1.36 | 1.20 | 1.03, 1.39 | 1.18 | 0.99, 1.38 | | 1.12 | 0.94, 1.33 |
| Difficult | 1.58 | 1.36, 1.82 | 1.64 | 1.41, 1.89 | 1.56 | 1.32, 1.84 | | 1.37 | 1.15, 1.63 |
| Very difficult | 2.06 | 1.73, 2.42 | 2.15 | 1.80, 2.53 | 2.05 | 1.67, 2.49 | | 1.56 | 1.25, 1.90 |
| Wealth, yen (ref, ≥50 million) | | | | | | | | | |
| 10 million–49.99 million | 1.09 | 0.98, 1.24 | 1.09 | 0.98, 1.23 | 1.07 | 0.94, 1.22 | | 1.03 | 0.90, 1.17 |
| 5 million–9.99 million | 1.16 | 1.04, 1.35 | 1.15 | 1.01, 1.32 | 1.13 | 0.97, 1.31 | | 1.04 | 0.87, 1.21 |
| 1 million–4.99 million | 1.35 | 1.21, 1.57 | 1.34 | 1.16, 1.54 | 1.32 | 1.12, 1.53 | | 1.21 | 1.02, 1.42 |
| <1 million | 1.49 | 1.31, 1.72 | 1.50 | 1.30, 1.73 | 1.40 | 1.19, 1.65 | | 1.20 | 1.001, 1.43 |
| Female |  | |  | |  | | |  | |
| Education, years (ref, ≥13) | | | | | | | | | |
| 10–12 | 1.10 | 1.00, 1.22 | 1.07 | 0.98, 1.18 | 1.04 | 0.94, 1.16 | | 1.01 | 0.89, 1.13 |
| <10 | 1.28 | 1.16, 1.40 | 1.18 | 1.08, 1.30 | 1.13 | 1.01, 1.26 | | 1.07 | 0.95, 1.20 |
| Past occupation (ref, professionals) | | | | | | | | | |
| White-collared workers | 1.01 | 0.90, 1.13 | 1.01 | 0.89, 1.14 | 1.03 | 0.90, 1.18 | | 1.07 | 0.91, 1.25 |
| Blue-collared workers | 1.16 | 1.04, 1.29 | 1.14 | 1.02, 1.28 | 1.14 | 0.998, 1.29 | | 1.11 | 0.96, 1.29 |
| Never worked before | 1.23 | 1.08, 1.39 | 1.11 | 0.96, 1.27 | 1.06 | 0.90, 1.24 | | 1.04 | 0.87, 1.24 |
| Income, yen (ref, ≥3 million) | | | | | | | | | |
| 2 million–2.99 million | 1.05 | 0.95, 1.16 | 1.03 | 0.93, 1.15 | 1.02 | 0.91, 1.15 | | 1.01 | 0.88, 1.15 |
| 1 million–1.99 million | 1.19 | 1.09, 1.30 | 1.17 | 1.06, 1.28 | 1.15 | 1.03, 1.28 | | 1.09 | 0.97, 1.23 |
| <1 million | 1.43 | 1.29, 1.57 | 1.38 | 1.25, 1.53 | 1.34 | 1.19, 1.51 | | 1.23 | 1.08, 1.41 |
| Subjective economic situation (ref, very comfortable) | | | | | | | | | |
| Comfortable | 1.10 | 0.98, 1.23 | 1.11 | 0.99, 1.24 | 1.18 | 0.94, 1.22 | | 1.06 | 0.91, 1.22 |
| Difficult | 1.38 | 1.23, 1.55 | 1.42 | 1.27, 1.60 | 1.56 | 1.16, 1.53 | | 1.22 | 1.05, 1.42 |
| Very difficult | 1.72 | 1.50, 1.97 | 1.78 | 1.55, 2.04 | 2.05 | 1.35, 1.88 | | 1.28 | 1.05, 1.55 |
| Wealth, yen (ref, ≥50 million) | | | | | | | | | |
| 10 million–49.99 million | 1.15 | 1.03, 1.29 | 1.14 | 1.01, 1.29 | 1.09 | 0.95, 1.24 | | 1.09 | 0.94, 1.27 |
| 5 million–9.99 million | 1.28 | 1.14, 1.44 | 1.27 | 1.11, 1.45 | 1.24 | 1.07, 1.44 | | 1.22 | 1.03, 1.43 |
| 1 million–4.99 million | 1.36 | 1.21, 1.54 | 1.32 | 1.15, 1.51 | 1.20 | 1.03, 1.41 | | 1.14 | 0.96, 1.35 |
| <1 million | 1.57 | 1.38, 1.78 | 1.55 | 1.35, 1.78 | 1.44 | 1.22, 1.69 | | 1.35 | 1.13, 1.62 |
|  | Model 1-b | | Model 2-b | | Model 3-b | | | Model 4-b | |
|  | PR | 95% CI | PR | 95% CI | PR | 95% CI | | PR | 95% CI |
| Age≥75 |  | |  | |  | | |  | |
| Education, years (ref, ≥13) | | | | | | | | | |
| 10–12 | 1.21 | 1.08, 1.34 | 1.16 | 1.04, 1.30 | 1.12 | 0.98, 1.26 | | 1.04 | 0.91, 1.19 |
| <10 | 1.36 | 1.23, 1.51 | 1.31 | 1.18, 1.45 | 1.23 | 1.10, 1.39 | | 1.15 | 1.01, 1.30 |
| Past occupation (ref, professionals) | | | | | | | | | |
| White-collared workers | 0.99 | 0.87, 1.12 | 0.96 | 0.84, 1.08 | 0.98 | 0.84, 1.12 | | 0.98 | 0.84, 1.14 |
| Blue-collared workers | 1.18 | 1.05, 1.32 | 1.15 | 1.03, 1.28 | 1.12 | 1.0001, 1.26 | | 1.08 | 0.94, 1.23 |
| Never worked before | 1.27 | 1.10, 1.47 | 1.14 | 0.98, 1.31 | 1.05 | 0.87, 1.25 | | 1.01 | 0.83, 1.24 |
| Income, yen (ref, ≥3 million) | | | | | | | | | |
| 2 million–2.99 million | 1.09 | 0.97, 1.23 | 1.09 | 0.97, 1.22 | 1.08 | 0.94, 1.23 | | 1.07 | 0.93, 1.24 |
| 1 million–1.99 million | 1.20 | 1.08, 1.34 | 1.20 | 1.08, 1.33 | 1.15 | 1.02, 1.29 | | 1.07 | 0.94, 1.22 |
| <1 million | 1.48 | 1.32, 1.65 | 1.43 | 1.28, 1.60 | 1.39 | 1.22, 1.59 | | 1.30 | 1.12, 1.51 |
| Subjective economic situation (ref, very comfortable) | | | | | | | | | |
| Comfortable | 1.16 | 1.02, 1.31 | 1.16 | 1.03, 1.33 | 1.13 | 0.98, 1.31 | | 1.06 | 0.90, 1.23 |
| Difficult | 1.47 | 1.29, 1.67 | 1.50 | 1.31, 1.71 | 1.40 | 1.20, 1.62 | | 1.14 | 0.96, 1.35 |
| Very difficult | 1.75 | 1.51, 2.03 | 1.76 | 1.52, 2.07 | 1.71 | 1.41, 2.05 | | 1.16 | 0.97, 1.38 |
| Wealth, yen (ref, ≥50 million) | | | | | | | | | |
| 10 million–49.99 million | 1.15 | 1.01, 1.30 | 1.15 | 1.01, 1.32 | 1.10 | 0.94, 1.26 | | 1.04 | 0.96, 1.13 |
| 5 million–9.99 million | 1.25 | 1.08, 1.45 | 1.24 | 1.07, 1.44 | 1.22 | 1.04, 1.42 | | 1.04 | 0.95, 1.14 |
| 1 million–4.99 million | 1.34 | 1.15, 1.53 | 1.33 | 1.15, 1.53 | 1.26 | 1.07, 1.49 | | 1.09 | 0.98, 1.20 |
| <1 million | 1.56 | 1.34, 1.81 | 1.54 | 1.32, 1.78 | 1.39 | 1.15, 1.65 | | 1.14 | 1.04, 1.26 |
| Age<75 |  | |  | |  | | |  | |
| Education, years (ref, ≥13) | | | | | | | | | |
| 10–12 | 1.06 | 0.98, 1.16 | 1.05 | 0.97, 1.15 | 1.05 | 0.96, 1.05 | | 1.01 | 0.91, 1.11 |
| <10 | 1.18 | 1.08, 1.29 | 1.17 | 1.07, 1.28 | 1.12 | 1.02, 1.23 | | 1.05 | 0.95, 1.16 |
| Past occupation (ref, professionals) | | | | | | | | | |
| White-collared workers | 0.98 | 0.88, 1.08 | 0.96 | 0.86, 1.07 | 0.97 | 0.87, 1.08 | | 0.98 | 0.87, 1.11 |
| Blue-collared workers | 1.14 | 1.04, 1.25 | 1.12 | 1.02, 1.23 | 1.11 | 1.0001, 1.22 | | 1.06 | 0.94, 1.18 |
| Never worked before | 1.14 | 0.95, 1.35 | 1.08 | 0.89, 1.28 | 0.99 | 0.80, 1.20 | | 0.98 | 0.77, 1.23 |
| Income, yen (ref, ≥3 million) | | | | | | | | | |
| 2 million–2.99 million | 1.03 | 0.93, 1.14 | 1.03 | 0.93, 1.13 | 1.03 | 0.92, 1.15 | | 1.00 | 0.88, 1.12 |
| 1 million–1.99 million | 1.22 | 1.11, 1.33 | 1.21 | 1.11, 1.32 | 1.20 | 1.09, 1.33 | | 1.14 | 1.03, 1.26 |
| <1 million | 1.49 | 1.33, 1.66 | 1.47 | 1.32, 1.63 | 1.40 | 1.23, 1.58 | | 1.22 | 1.06, 1.40 |
| Subjective economic situation (ref, very comfortable) | | | | | | | | | |
| Comfortable | 1.10 | 0.96, 1.25 | 1.10 | 0.97, 1.26 | 1.08 | 0.92, 1.24 | | 1.08 | 0.91, 1.26 |
| Difficult | 1.46 | 1.28, 1.67 | 1.48 | 1.30, 1.69 | 1.40 | 1.20, 1.63 | | 1.27 | 1.07, 1.49 |
| Very difficult | 1.97 | 1.68, 2.28 | 1.98 | 1.71, 2.28 | 1.76 | 1.47, 2.09 | | 1.37 | 1.12, 1.66 |
| Wealth, yen (ref, ≥50 million) | | | | | | | | | |
| 10 million–49.99 million | 1.09 | 0.98, 1.22 | 1.09 | 0.98, 1.22 | 1.08 | 0.95, 1.22 | | 1.06 | 0.93, 1.20 |
| 5 million–9.99 million | 1.19 | 1.04, 1.34 | 1.19 | 1.04, 1.35 | 1.17 | 1.0003, 1.35 | | 1.11 | 0.95, 1.28 |
| 1 million–4.99 million | 1.34 | 1.17, 1.53 | 1.34 | 1.16, 1.52 | 1.27 | 1.09, 1.47 | | 1.19 | 1.01, 1.39 |
| <1 million | 1.52 | 1.32, 1.73 | 1.53 | 1.33, 1.74 | 1.42 | 1.21, 1.66 | | 1.26 | 1.06, 1.48 |

Abbreviations: PR, prevalence ratio; 95 % CI, 95% credible interval.

Severe low back pain was defined as low back pain leading to limitations in daily life.

Socioeconomic status was separately added to each model.

Model 1-a, a crude model; Model 2-a, with age adjusted to Model 1; Model 3-a, with number of persons living together, marital status, musculoskeletal disease, BMI, drinking habit, smoking, physical activity, and municipalities added to Model 2; Model 4-a, with depression added to Model 3-a.

Model 1-b, a crude model; Model 2-a, with sex adjusted to Model 1; Model 3-b, with number of persons living together, marital status, musculoskeletal disease, BMI, drinking habit, smoking, physical activity, and municipalities added to Model 2; Model 4-b, with depression added to Model 3-b.

**Table S6.** Differences in medical access for low back pain among participants having low back pain by socioeconomic status (n = 15,401).

| Socioeconomic status | Limited | Unlimited | *P*-value |
| --- | --- | --- | --- |
|  | N (%) | N (%) |  |
| Educational attainment, years |  |  |  |
| <10 | 3,389 (52.1) | 3,110 (47.9) | <.01 |
| 10–12 | 2,502 (45.1) | 3,043 (54.9) |  |
| ≥13 | 1,210 (42.7) | 1,627 (57.3) |  |
| Past occupation |  |  |  |
| Professionals | 942 (43.5) | 1,222 (56.5) | <.01 |
| White-collared workers | 1,395 (44.5) | 1,742 (55.5) |  |
| Blue-collared workers | 2,821 (47.5) | 3,121 (52.5) |  |
| Never worked before | 453 (55.4) | 365 (44.6) |  |
| Equivalized household income, yen |  |  |  |
| <1 million | 1,130 (54.4) | 946 (45.6) | <.01 |
| 1 million–1.99 million | 2,232 (47.0) | 2,517 (53.0) |  |
| 2 million–2.99 million | 1,253 (44.5) | 1,561 (55.5) |  |
| ≥3 million | 1,229 (43.3) | 1,610 (56.7) |  |
| Subjective economic situation |  |  |  |
| Very difficult | 710 (52.5) | 643 (47.5) | <.01 |
| Difficult | 2,633 (48.0) | 2,853 (52.0) |  |
| Comfortable | 3,163 (46.5) | 3,634 (53.5) |  |
| Very comfortable | 574 (47.6) | 632 (52.4) |  |
| Wealth, yen |  |  |  |
| <1 million | 725 (51.3) | 687 (48.7) | <.01 |
| 1 million–4.99 million | 911 (51.0) | 874 (49.0) |  |
| 5 million–9.99 million | 915 (46.8) | 1,039 (53.2) |  |
| 10 million–49.99 million | 2,028 (43.5) | 2,630 (56.5) |  |
| ≥50 million | 681 (43.3) | 893 (56.7) |  |

Chi-squared test was performed.
